# The Role of Pharmacological Treatment in the Chemoreflex Modulation

**DOI:** 10.3389/fphys.2022.912616

**Published:** 2022-06-14

**Authors:** Anna Langner-Hetmańczuk, Stanisław Tubek, Piotr Niewiński, Piotr Ponikowski

**Affiliations:** ^1^ Institute of Heart Diseases, Wroclaw Medical University, Wroclaw, Poland; ^2^ Institute of Heart Diseases, University Hospital, Wroclaw, Poland

**Keywords:** chemoreflex, peripheral chemoreceptors, chemoreceptors sensitivity, chemoreceptors tonicity, pharmacotherapy, medications

## Abstract

From a physiological point of view, peripheral chemoreceptors (PCh) are the main sensors of hypoxia in mammals and are responsible for adaptation to hypoxic conditions. Their stimulation causes hyperventilation—to increase oxygen uptake and increases sympathetic output in order to counteract hypoxia-induced vasodilatation and redistribute the oxygenated blood to critical organs. While this reaction promotes survival in acute settings it may be devastating when long-lasting. The permanent overfunctionality of PCh is one of the etiologic factors and is responsible for the progression of sympathetically-mediated diseases. Thus, the deactivation of PCh has been proposed as a treatment method for these disorders. We review here physiological background and current knowledge regarding the influence of widely prescribed medications on PCh acute and tonic activities.

## Introduction

The clusters of chemoreceptor cells in the human body are localized in the central nervous system—central chemoreceptors (CCh) and in the carotid (CB) and aortic bodies - peripheral chemoreceptors (PCh) (O'Regan and Majcherczyk, 1982). The former are mainly hypercapnia sensors while the latter are activated by hypoxia, hypercapnia and acidosis (O'Regan and Majcherczyk, 1982). Activation of both areas leads to hyperventilation, sympathetically-mediated vasoconstriction and usually tachycardia (Kara et al., 2003; Tubek et al., 2018; Zera et al., 2019). The magnitude of the acute (provoked) physiological response (most commonly ventilatory response) to the stimulation of PCh is called peripheral chemoreceptors sensitivity (PChS) and is used as the measure of PCh function (Tubek et al., 2018). However, emerging experimental evidence indicates that PCh exert also tonic (independent of the stimuli) influence on cardio-respiratory centers, leading to a chronic increase in sympathetic tone (Sinski et al., 2012; Tubek et al., 2018; Tubek et al., 2021). This influence is neglectable in healthy individuals; however, it is significantly increased in patients with hypertension (HT), heart failure (HF) or obstructive sleep apnoea and was recognized as an important factor in the pathophysiology and progression of these diseases (Schultz et al., 2007; Sinski et al., 2012; Paton et al., 2013a; Schultz et al., 2013; Xing et al., 2014). The most widely used measurement employed to assess the PCh tonic activity (PChT) is the magnitude of the ventilatory or sympathetic nerve activity (SNA) drop following PCh inhibition (Schultz et al., 2007; Tubek et al., 2018).

Several molecular mechanisms have been identified as potential PChS and PChT sensitizers including upregulation of angiotensin II/nicotinamide adenine dinucleotide phosphate (NADPH) oxidase and serotonin/NADPH oxidase; downregulation of nitric oxide synthases (NOS) and glucagon-like peptide-1 receptor or increase in leptin, endothelin, or proinflammatory cytokines concentrations (Chen et al., 2002; Li et al., 2005; Li et al., 2007; Schultz et al., 2007; Yuan et al., 2018; Shin et al., 2019; Iturriaga et al., 2022; Pauza et al., 2022). Moreover, numerous modulatory neurotransmitters have been identified in CB, such as adrenaline, noradrenaline, dopamine, adenosine and acetylcholine, which adjust PChs’ activity to current needs (Pérez-García et al., 1993; Prabhakar, 1994).

CB has been proposed as a therapeutic target in sympathetically mediated disease based on animal studies (Abdala et al., 2012; Del Rio et al., 2013; McBryde et al., 2013; Schultz et al., 2013) and even some encouraging data from humans, regarding interventional CB deactivation, has been published (Narkiewicz et al., 2016; Niewinski et al., 2017). However, such procedures, particularly bilateral, may be complicated with sleep apnoea exaggeration or inappropriate adaptation to hypoxic environmental conditions e.g. during plane flights (Tubek et al., 2018; Niewinski et al., 2021). This along with invasive and irreversible character of CB excision encourages to look for pharmacological methods of PCh modulation.

Bearing in mind that at least some of the mechanisms leading to PCh overfunctionality are covered with guidelines-recommended cardiovascular medications we review the available literature to: 1) find out proved and potential interactions between these drugs and PCh function to present possible ways of pharmacological modulation of chemoreflex, 2) expose gaps in the evidence of such interaction to stimulate further studies in the field, 3) stress the role of the therapy individualization—drugs decreasing PCh function should be preferred in a patient with known PCh overfunctionality.

### Adrenergic Drugs

Dopaminergic and both alpha- and beta-adrenergic receptors are present in chemosensory (type I) glomus cells of most mammals species (Kou et al., 1991; Pérez-García et al., 1993; Prabhakar, 1994). Moreover, the release of dopamine and adrenaline from chemosensory cells in the response to hypoxia is well described in rodents (Fidone et al., 1982; Gonzalez et al., 1992; Kato et al., 2013). According to animal studies, alpha_2_-receptors agonists seems to have an inhibitory influence on CB, when beta-receptors agonists (βRA), most likely β_2_RA, are enhancing the CB-mediated response (Mir et al., 1983; Kou et al., 1991). On the other hand, the response to dopamine is dose-dependent. Low-dose dopamine infusion (LDDI) affects mostly type 2 dopamine receptors (D_2_R) on chemosensory cells, which in consequence limits the release of neurotransmitters (Lehmann et al., 1983; Prabhakar, 1994; González et al., 1995). High doses of dopamine activate both D_1_R and D_2_R in CB, but the stimulating effect of postsynaptic D_1_R dominates and leads to increased stimulation of nerve endings surrounding CB (González et al., 1995). According to the published evidence, it seems that the crucial physiological role of catecholamines in CB is self-limitation of the amplitude and persistence of the response to acute stimulation (PChS modulation) (Prabhakar, 1994). Nevertheless, it was shown recently, that the pre-treatment with propranolol (non-selective beta-blocker) prevents the development of chronic intermittent hypoxia (IH) induced increase in PChT (Alzahrani et al., 2021). It should be finally mentioned, that adrenergic drugs exerts also direct vasomotor effects modulating the perfusion of CB, which may influence the organ functionality (Iturriaga et al., 2016). Since, the net effect of systemically administered adrenergic drug on PCh would be dependent on the pattern of adreno-receptor interactions on chemosensory cells and chemosensory organs perfusion changes.

#### Beta-Receptors Agonists

In humans, the influence of βRA on PCh is well documented. Similarly as in animals oral, single dose, administration of fenoterol (β_2_RA) increased baseline ventilation by 15%, as well as, augmented ventilatory response to both hypoxia and hyperoxic hypercapnia in healthy subjects (Yoshiike et al., 1995). This finding indicates that β_2_RA modulates both central and peripheral chemoperception. Intravenous infusion of another β_2_RA—salbutamol—increased ventilatory responses to hypoxic hypercapnia (stimulation of both PCh and CCh) and hyperoxic hypercapnia (isolated CCh stimulation) (Leitch et al., 1976).

Effects of β_1_RA—isoprenaline—were described by Heistad et al. in healthy individuals. The authors revealed a stimulating effect of this catecholamine on resting ventilation, which was reversed with a non-selective beta-receptors blocker (βRB)—propranolol and well known PCh inhibitor—100% hyperoxia (Heistad et al., 1972). However, isoprenaline did not exaggerate the ventilatory response to hypoxia. Due to these difficult to interpret results, which were in contrast to the animal data (Folgering et al., 1982), an elegant study in goats was designed by Hudgel et al. Goats with intact and denervated CB underwent the same protocol consisting of isoprenaline administration and isocapnic, hypoxic exposures (Hudgel et al., 1986). The study showed, that isoprenaline caused the same resting hyperventilation in both groups. This response was reversed by propranolol. Further, despite an increase in minute ventilation, isoprenaline did not alter the carotid sinus nerve chemoreceptor discharge rate during hypoxia. Therefore, despite β_1_RA increases resting ventilation the influence of these molecules on PChS is scarce both in animals and humans.

Intravenous administration of non-selective β1-, β2- and α1-adrenergic agonist—dobutamine—caused a dose-dependent increase in resting minute ventilation and increased ventilatory and sympathetic responses to hypoxia (PChS), but not to hyperoxic hypercapnia (CChS) in healthy (Velez-Roa et al., 2003; Pathak et al., 2006). The effects of dobutamine were blunted in five subjects with atenolol—a moderately selective β_1_RA, which despite higher affinity to β_1_R binds also to β_2_R (Smith and Teitler, 1999; Pathak et al., 2006). Taking into account the neglectable effects of isoprenaline (β_1_RA) on hypoxia-induced responses it seems that β_2_RA and α_1_RA components are responsible for dobutamine’s ventilatory actions.

#### Beta-Receptors Blockers

Beloka et al. found that 7 days of oral beta-blocked with selective β_1_-receptor blocker (β_1_RB) bisoprolol did not change baseline ventilation; ventilatory and sympathetic nervous system (measured with muscle SNA) responses to hypoxia or hypercapnia in healthy (subjects with generally low PChS and PChT) (Beloka et al., 2008).

However, in the study in HF patients (subjects with generally high PChS and PChT) treatment with carvedilol (non-selective β_1_RB, β_2_RB, α_1_RB), reduced resting ventilation (during normoxia and hypoxia) and exercise hyperventilation compared to placebo (Agostoni et al., 2006). Unfortunately, PChS was not assessed in the study. But in another study in HF population—CARNEBI trial—2-months long treatment with carvedilol caused a significant decrease in PChS and central chemosensitivity compering to the treatment with bisoprolol (Contini et al., 2013). Moreover, PChS but not CCh sensitivity was also diminished with nebivolol compared to bisoprolol (Contini et al., 2013). The actions of bisoprolol on PCh were not assessed against placebo.

Hence, it seems that both peripheral and central chemoperception in humans are modulated by β_2_R but not β_1_R. Otherwise, nebivolol β_1_RB, which is also β_3_-agonist, seems to decrease PChS via the nitric oxide (NO) pathway. Therefore non-selective or NO-active beta-blockers should be preferred, when PChS modulation is aimed. This observation is particularly interesting in the contest of the recently described protective mechanism of propranolol (non-selective β-blocker) on chronic IH induced hypertension in rats (Alzahrani et al., 2021).

#### Alfa-Receptors

Otherwise, data regarding the influence of αR stimulation or inhibition on PCh is scarce. Heistad et al. administered phenylephrine (α_1_RA) in healthy individuals, which decreased resting ventilation (Heistad et al., 1972). However, the influence of the drug on hypoxic ventilatory response was not tested, since the role of PCh or CCh in this change remains questionable.

#### Dopamine

The influence of dopamine on PCh is well described and is known to be dose-dependent (Iturriaga and Alcayaga, 2004). In healthy humans, high-dose infusion increased and LDDI decreased hypoxic minute ventilation (Welsh et al., 1978). Apart from diminishing the ventilatory response to acute hypoxia (PChS), LDDI diminished also arterial pressure response to the stimuli (Niewinski et al., 2014). Some evidence has also been found for the influence of LDDI on PChT—namely, the infusion decreased normoxic minute ventilation in HFrEF (patients with presumed high PChT), but the data in healthy is inconsistent (with presumed low PChT) (Welsh et al., 1978; Van De Borne et al., 1998; Niewinski et al., 2014). Moreover, withdrawal of LDDI causes temporary hyperventilation probably due to the sudden release of accumulated neurotransmitters following PCh re-activation (Niewinski et al., 2014). LDDI seems to be a good inhibitor of both PChS and PChT for human experimental use; however, one has to be aware, that: 1) high interindividual variability in the inhibitory effect magnitude is observed (Limberg et al., 2016) and 2) the medication itself exerts some hemodynamic effects including slight tachycardia and vasodilatation. The latter are most likely secondary to D_2_R and adrenergic β_1_R activation, which may mask the hemodynamic effects of PChT inhibition (Van De Borne et al., 1998; Overgaard and Dzavík, 2008; Niewinski et al., 2014).

### Renin-Angiotensin-Aldosterone Axis Modulators

The presence of angiotensinogen and expression of both angiotensin receptors (ATR1 and ATR2) has been confirmed in CB (Lam and Leung, 2002; Leung et al., 2003). Generally, the activation of the ATR1 and ATR2 generates opposite physiological effects at CB level; however, the stimulatory effects of ATR1 dominate the final response (Schultz, 2011). Overexpression of these receptors, leading to augmentation of PChS, has been identified in animal models of IH and HF (Li et al., 2006; Fung, 2014; Lam et al., 2014). Moreover, administration of ATR1 inhibitor in HF rabbits decreased PChS and renal SNA response to hypoxia; however, neither resting ventilation, nor resting renal SNA were changed—lack of evidence on PChT modulation (Li et al., 2006). The similar manoeuvre did not change PChS in healthy animals. Otherwise, administration of angiotensin II analogs increased ventilatory and renal SNA responses to hypoxia (acute PChS) in healthy animals, but not in HF ones (Li et al., 2006). These data suggest that angiotensin signalling has a modulatory role in PChS and depends on the expression and saturation of ATR1. It should be noticed, that angiotensin II acts also directly on central nervous system leading to systemic sympathoexcitation, which via efferent nerve fibers may further modulate PCh functionalities (Ye et al., 2002b).

The influence of ATR1 inhibition on PCh is poorly studied in humans. Among a few studies in the topic, all tested selective ATR1 blocker—losartan. Consistently with animal data administration of the drug did not affect resting ventilation (PChT) in healthy individuals (Foster et al., 2010) and in patients with obstructive sleep apnoea (OSA) (Morgan et al., 2018). Otherwise, the study conducted in hypertensive patients with OSA (with presumed high PChS) revealed a lack of the influence of losartan on the hemodynamic, muscle SNA and ventilatory responses to hypoxia (PChS) (Morgan et al., 2018). However, patients included were optimally treated (majority were prescribed with continuous positive airway pressure—CPAP) and had generally low PChS and muscle SNA response to hypoxia at the study entry, what finally influenced on negative results of the study (Morgan et al., 2018). Further, Foster et al. (2010) found that treatment with losartan prevents IH-induced increase in mean arterial pressure in healthy individuals; however, were unable to prove the influence of the drug on PChS since IH in their model did not increase PChS. Unfortunately, the influence of ATR antagonists in other populations of patients with presumed high PChS and PChT, like HF or HT, has not been tested. Solaiman et al. (2014) tested the effects of intravenous infusion of low and high doses of angiotensin II in healthy volunteers, but in contrast to animal studies neither peripheral nor central chemosensitivity was influenced with the intervention. The influence of angiotensin-converting enzyme inhibitors and aldosterone antagonists on PChS has not been tested in animal and human subjects.

### Nitric Oxide

Both enzymes responsible for nitric oxide (NO) synthesis namely neuronal and endothelial synthases are present in CB (Wang et al., 1993). Two modes of NO synthesis have been distinguished in rodents’ CBs: 1) basal production, which is responsible for limiting CB tonic discharges during normoxia (Ding et al., 2008; Ding et al., 2011) and 2) hypoxia-dependent acute/provoked, which is self-limiting the acute response (Ye et al., 2002a; Yamamoto et al., 2006; Fung, 2014). The intensity of the basal/chronic NO synthesis acts on PChT—taking an important role e.g. in the upregulation of this parameter in HF (Ding et al., 2008) when the provoked production modulates PChS diminishing the acute response to hypoxia, e.g. in chronic IH model (Ye et al., 2002a).

The influence of NO concentrations on PChS in humans was tested by Bock et al. Researchers revealed that long-term dietary nitrate supplementation with beetroot powder in older healthy subjects caused a significant reduction in PChS compared to placebo (Bock et al., 2017a; Bock et al., 2017b). Further, Notay et al. found a drop in muscle SNA following acute beetroot oral supplementation in healthy humans, which may be hypothetically the consequence of PChT inhibition; however this needs to be confirmed in future studies (Notay et al., 2017). Our group is currently investigating the influence of low-dose intravenous NO (which is used to treat patients with acute HF decompensation), on PChS in stable HF patients and the results will be available soon.

### Antiplatelets—P2Y12 Adenosine Receptors Antagonists

Storage of adenine nucleotides was described in CB cells in animals and adenosine, via activation of postsynaptic P2X and A2s receptors in the CBs, is one of the neurotransmitters responsible for excitatory response to hypoxia (Böck, 1980; Leonard et al., 2018). However, adenosine acts also on the presynaptic P2Y receptors group, including P2Y12 receptors, which inhibit type I cells by negative feedback mechanisms and limits the response (Agarwal et al., 2011; Leonard et al., 2018). Intracarotid injections of adenosine in cats caused a dose-dependent increase in the discharge rate of CB neural afferents (McQueen and Ribeiro, 1983). The physiological role of adenosine in CB has also been confirmed in conscious hypertensive humans, where unilateral intracarotid administration of adenosine caused dose-dependent hyperventilation and hypertension (Tubek et al., 2016). Pharmacological modification of CB has been tested by Pijacka et al. in spontaneously hypertensive rats. Administration of P2X3 antagonist normalized tonic sympathetic activity to the extent seen in healthy animals and reduced chemoreflex-mediated sympathetic activity and additionally decreased blood pressure and heart rate (Pijacka et al., 2016). Overexpression of P2X3 receptors was also found in hypertensive humans cadavers, which suggests a possible role of these structures in the pathogenesis of human hypertension (Pijacka et al., 2016). This, so far hypothetical role, may get investigatable soon, as gefapixant - P2X3 receptors antagonist - is currently tested in humans as a drug for chronic cough (Muccino et al., 2020).

PCh functionality may be theoretically influenced by antiplatelet drugs widely prescribed in humans—clopidogrel, tikagrelor or prasugrel—via adenosine P2Y12 receptor inhibition. Moreover, one of the widely prescribed P2Y12 inhibitors—ticagrelor—has proven, both in animal and human models, inhibitory influence on adenosine cellular uptake, leading to increased tissue adenosine concentrations (Cattaneo and Faioni, 2012). Further, some clinical evidence may indicate possible effects of ticagrelor on chemoreflex. First of all, the most common side effect of ticagrelor treatment is dyspnea, which was hypothesised to be mediated by chemoreceptors (Cattaneo and Faioni, 2012). Secondly, exaggeration/new onset of disordered breathing (including Cheyne-Stokes respiration) was observed in patients following ticagrelor administration (Giannoni et al., 2016; Giannoni et al., 2021). Giannoni et al. tested chemoreflex response in the group of patients with such a complications and found higher ventilatory response to hypercapnia (central chemosensitivity) compared to patients without these side effects (Giannoni et al., 2021). Similarly, higher central chemosensitivity was found in the whole group of patients treated with ticagrelor compared to patients on prasugrel (Giannoni et al., 2021). The study aiming to reveal the influence of ticagrelor on PChS is being currently conducted in our center.

### Diuretics

The only diuretic drug with a confirmed influence on chemoreflex is acetazolamide. This carbonic anhydrase inhibitor leads to mild metabolic acidosis with a consequent: 1) ventilatory compensation (drop in CO_2_ arterial levels) and 2) increase in cerebral blood flow. Net effects of these changes are: desensitization of PChS and augmentation of central chemoreflex (the ventilatory response to carbon dioxide) (Adams and Johnson, 1990; Wagenaar et al., 1998; Vovk et al., 2000; Fontana et al., 2011). Moreover, it has been recently demonstrated that administration of acetazolamide diminishes periodic breathing and improves haemoglobin saturation in HF patients (Fontana et al., 2011; Javaheri et al., 2014). The effects of loop, thiazide and thiazide-like diuretics on peripheral chemoreceptors have not been studied.

### Calcium Channel Blockers

The transient influx of calcium ions into glomus type I cells of CB is crucial for the release of neurotransmitters in hypoxic conditions (Shirahata and Fitzgerald, 1991). In an animal model, verapamil administration attenuated resting ventilation (PChT) and PChS in anesthetized rats but did not influence central chemosensitivity (Chapman, 1985). However, this was not confirmed in healthy humans by Long et al. (1989). Another study in healthy humans by Watt et al. revealed no changes in resting minute ventilatory in normoxic and hypoxic hypocapnic conditions following amlodipine vs placebo administration (Watt et al., 2000). These data suggest that calcium-blockers have no influence on PChS in healthy subjects, but further studies in patients with presumed high PCh activity have to be performed.

### Digitalis Glycosides

Digitalis glycosides affect intracellular calcium level indirectly by inhibiting Na+/K+ ATPase leading to hypernatremia and enhanced calcium ions influx (Levi et al., 1994). Animal data revealed that intravenous infusion of oubain during normoxia causes a transient increase in discharge rate in the carotid sinus nerve innervating the cat’s CB (McQueen and Ribeiro, 1983). In the same experiment, oubain led also to increased hypoxic CB activity. In healthy humans, digoxin and cedilanid were found to increase PChS and did not affect hypercapnic response which suggest the influence only on PCh (Schobel et al., 1994; Janssen et al., 2010). Some indirect evidence may also indicate an influence of digoxine on PChT. Pagenelli et al. found that the administration of the drug abolishes hyperoxia induced decrease in HR and cardiac output in HF patients. These effects were linked with PChT inhibition by the authors; however, this interaction has to be verified in the future trials (Paganelli et al., 2001).

### Anti-Inflamatory Medications and Anti-Oxidants

The modulatory role of proinflammatory cytokines on CB functionality was revealed in animal models (Iturriaga et al., 2022). Both local inflammation (within CB) and systemic inflammatory response are increasing PChT and PChS in rats (Iturriaga et al., 2022; Katayama et al., 2022). Ibuprofen, which is well-known cyclooxygenase 1 and 2 inhibitor suppresses proinflammatory cytokines production (Scheuren et al., 1998) and prevents chronic hypoxia aftereffects such as: 1)increased interleukin-1 and tumour necrosis factor alfa production within CB, 2) hypertension, 3) enhanced hypoxic ventilatory response (PChS) and carotid body discharges (PChT) (Liu et al., 2009; Del Rio et al., 2012).

Another mechanism enhancing CB functionality following IH is oxidative stress. In rats, ascorbic acid added to drinking water reduced the malondialdehyde and 3-nitrotyriosine plasma (oxidative stress markers) levels and prevented IH-induced augmentation of PChS (Del Rio et al., 2010).

The data regarding the influence of anti-inflammatory and antioxidants on PChS and PChT in humans is missing.

## Discussion

Molecules with confirmed or suspected modulatory influence on PCh are listed in Table 1. However, despite the treatment the prevalence of elevated PCh functionality in sympathetically mediated diseases is still high - e.g. in HF patients PCh oversensitivity can be identified in more than every third patient despite guidelines-recommended treatment including beta-blockers and renin-angiotensin-aldosterone axis modulators (Niewinski et al., 2013). Thus, the development of the novel methods for chemoreflex modulation, preferably these reversible and titerable, is required. As it was presented above the complexity of PCh function, numerous, probably not completely discovered, pathophysiological pathways leading to PCh overfunctionality and numerous modulatory factors has to be considered when designing future research in the field. First of all, PChT rather than PChS or both should be aimed with the treatment to limit permanently increased SNA (Paton et al., 2013b; Paton et al., 2013a; 61). Secondly, the effects of PChT modulation can be found usually only in subjects with excessive PCh output (in healthy the input from PChT is neglectable), thus the subjects with presumed high PChT should be included in the trials. Thirdly, the pathomechanism that triggers PCh overactivity may differ across diseases and, therefore, potential treatments to reduce PChT and/or PChS are likely to be disease-specific (Schultz et al., 2007). Fourthly, vasodilatory agents will increase SNA via baroreceptors unload, hence PChT (with SNA dynamics) should be assessed after the restoration of baseline blood pressure or should be expressed as changes in minute ventilation (Ding et al., 2008). Fifthly, PChS or PChT are dynamic parameters, which change within particular individuals allowing adaptation to environmental or functional changes, e.g. during physical activity (Weil et al., 1972); since, the effects of novel PCh modulators should be tested both at rest and during physiological challenges relevant to patients’ daily life. Finally, the novel treatment techniques developed in animal models (medication-naïve subjects), may not work in human patients, because the mechanisms addressed may be already covered with prescribed drugs.

**TABLE 1:** Confirmed and potential (based on animal data) effect of medications on chemoreceptors functionality.

Active Substance	Physiological Mechanism of interaction with PCh	Effect on PChS	Effect on PChT	Effect on CCh Sensitivity
fenoterol, salbutamol	activation of β_2_R on type 1 glomus cells	↑	**?**	↑
isoprenaline	activation of β_1_R	↔	↔	**?**
dobutamine	activation of β_1_R, β_2_R, α_1_R	↑	**?**	↔
bisoprolol	blockade of β_1_R	↔*	↔*	↔*
carvedilol	blockade of β_1_R, β_2_R, α_1_R	↓	**?**	↓
nebivolol	blockade of β_1_R and increased NO synthesis	↓	**?**	↔
phenylephrine	activation of α_1_R	**?**	**?**	**?**
dopamine	dose-dependent activation of D_1_R, D_2_R, α_1_-R and β_2_-R	Low dose:	Low dose:	↔
		↓	↓	
		High dose:	High dose:	
		**?**	**?**	
losartan	blockade of ATR1	↓	↔	**?**
NO oral supplementation	suspected—increased perfusion of the PCh	↓	↓	↓
ticagrelor	inhibition of adenosine P2Y12 receptors and cellular adenosine uptake	**?**	**?**	**?**
acetazolamide	metabolic acidosis with consequent hypocapnia	↓	**?**	↑
verapamil, amlodipine	blockade of calcium ions influx into glomus type 1 cells	↔*	**?**	↔
digoxin	increased calcium ions intracellular concentration	↑*	Suspected ↓	↔*
ibuprome	cyclooxygenase 1 and 2 inhibition	↓ (?)	↓	**?**
leptin	activation of Trpm7	**?**	↑	↑
endothelin	activation of endothelin receptor type A and B	↔ (Gujic et al., 2007)	↑	**?**
cytokines	activation of IL-1β, IL-6, TNF-α receptors	↑ (?)	↑	**?**

↑, medication studied in humans, causes augmentation of the parameter; **↑**, medication studied only in animals, causes augmentation of the parameter; ↓, medication studied in humans, causes reduction of the parameter; **↓**, medication studied only in animals, causes reduction of the parameter; ↔, medication studied in humans, has neglectable influence on the parameter; **↔**, medication studied only in animals, has neglectable influence on the parameter; **?**, no data available; *, drug studied only in healthy humans, the influence on PCh need to be studied in diseased subjects; αR,alfa-adrenergic receptors; ATR, angiotensin receptors; βR, beta-adrenergic receptors; NO, nitric oxide; PCh, peripheral chemoreceptors; PChS, Peripheral Chemoreceptors Sensitivity; PChT, Peripheral Chemoreceptors Tonic Activity; CCh, Central Chemorecoptors; Trpm7, transient receptor potential melastatin 7; IL, interleukin; TNF, tumour necrosis factor.
